# Erie County Medical Society

**Published:** 1887-02

**Authors:** 


### *ERIE COUNTY MEDICAL SOCIETY.*

The annual meeting of the Society was held January 11, 1887, at Y. M. C. A. building. There was a goodly number in attendance. Dr. Thornton read the minutes of the last meeting, which were approved.

Dr. Dorland, the President, addressed the Society as follows:

PRESIDENT'S ADDRESS.

Custom as well as our law demands as one of the duties of the expiring presiding officer, that he should leave a legacy of written words behind him. In compliance with this duty, I propose to read a very short paper, which, whatever else it may contain, will assuredly not interpret duly the sentiment of him who wrote it, unless it make you sensible of his grateful and kindly feelings for the unmerited honor your courtesy has conferred upon him, and for the invariable consideration and forbearance extended to him while in the discharge of his official duties. For all which I can but simply thank you, and proceed without further preamble with the reading of the paper. It would ill become the writer on such an occasion as this to detain you with a lengthy paper, though the subject might be ever so interesting, and the writer ever so competent, which he with becoming modesty disclaims. My compulsory paper is on the subject of drugs in the treatment of disease. I am aware that much has been said and written upon the subject of excessive medication, which is largely due, I suppose, to the great multiplication of remedies, and in part, I believe to the weakness of many in catering to the morbid propensity of patients for drugs. Still notwithstanding the sentient words of protest that have been uttered against this empirical practice, I am frequently cognizant of indulgences in it by men of good and regular standing to an extent in my judgment hardly compatible with the seriousness and gravity of our calling. I assume that among intelligent, well-informed, conscientious physicians, there is less faith today in drugs in the treatment of disease than at any previous time since the science of medicine took on the character of a learned profession. The progress of true medical science has greatly qualified our estimate of the value of mere medicine in the treatment of disease. Every day is showing the value of measures founded upon a rational study of the body and its diseases; at the same time, no sound man is very sanguine in his expectations of discovering specifics. In proof of the statement that with the advance of medical knowledge, faith in drugs *per se* has diminished, I may note that wounds that used to be treated with unguents containing twenty ingredients, more or less, are found to do far better with simple water dressings. The pneumonia that used to be attacked with heroic remedies, bleeding, antimony and calomel, now gets well with horizontal position and dover powder. Typhoid fever instead of being drugged, is now treated with good air, pure milk, and plenty of water, both internally and externally. The chief characteristic of advancing therapeutics is to respectfully watch the natural course of disease, to regard pathological processes only as modifications of physiological ones, with natural tendency to terminate in harmonious and healthy action when the obstructions are overcome which these pathological processes themselves were put in action to remove. All this is done without any detraction from the dignity and importance of the physician. He is, indeed, much more worthy of public admiration and confidence than he who would attain no more than the same result by the most active medical warfare. True physicians never talked so modestly as now about curing disease. Pseudo medicine, on the contrary, talks loudly of specifics, and makes no account of Nature's provisions for the cure of physical ills. With them, medicine is everything; Nature, perverse and destructive. One medicine with our medicine monger is never sufficient to cure disease; it always requires two or more, alternated with exactness and taken frequently. Upon this plan the poor victim of the infinitisimal often becomes the receptacle of a most unprincipled amount of medicine. In extenuation of this practice, it is often said that the people are not sufficiently educated to accept the advice of a physician without medicine. This is in some degree true, but sensible patients will soon learn to prize and properly appreciate the advice of an honest physician. The habit of giving medicine in all cases, and from two or three to half a dozen at a time often, has become an established practice with very many practitioners. While I would not assert that the compounds given are usually very injurious, or in anyway capable of doing great harm, I would maintain that they are unnecessary, sometimes injurious, and always objectionable, unless plainly indicated. It requires much less time and thought to write a prescription than it does to explain why no prescription is necessary. Many persons at first might not be as well satisfied with little or no medicine, but in the end their opinion of the physician who would not unnecessarily medicate, would be more exalted. I insist that by many, (though by less I believe than formerly,) disease is greatly over-treated. It did appear at one time that the vagaries of Hahnnemann would do something to check the abuse of medicine, but even the belief that homeo pathic remedies would at least do no harm, has long since been dissipated with the knowledge that even the disciples of this transparent delusion are drugged more extensively and often more dangerously than any other. I would not by any means say aught that would lessen faith in judicious medication, or detract in the least from the confidence which the intelligent physician reposes in his therapeutics, but I would rather increase his expectation of usefulness by excluding from his armament the useless weapons and suggesting the employment of the remaining only when necessity demands. That there is, however, on the part of many, far too much confidence in medicine, and too great readiness to attribute results to its effects, I firmly believe. This holds good both with physicians and patients, but whatever of censure rightfully attaches to it, should apply to the physicians, for with the laity we can expect no intelligent discrimination when we consider the marvelous ignorance displayed by a large part of the community in regard to the profession of medicine, which great ignorance physicians know full well has even furnished a rich field of operations for every species of medical humbuggery; richer by far, I believe, than the gold mines of California, or the silver mines of Nevada. More careful study of the natural history of disease, and more thorough acquaintance with the conditions of its progress and decline, will enable us to more correctly estimate the true value of remedies, and prevent in great measure the administration of medicines which can do nothing better than amuse the patient while nature cures the disease. I am aware that those of my hearers who are fully abreast of the times in regard to modern pathology, will likely think that such a paper as I present would have been more appropriate twenty years ago; that with the marvelous light the germ theory has and is throwing upon the pathology of to-day, the science of therapeutics must of necessity assume more of intelligence and precision—a result most devoutly to be wished for. But until there is less of the senseless and unreasonable dosing which we know still obtains, there is excuse, I think, for some such strictures as my paper very imperfectly presents.

The following statement (with which I close my paper) was made several years ago by a prominent journalist, and, though rather ancient, contains truths I think that are pertinent to the present as well as to the past days of medical history. It is as follows: "To reform and educate the masses to a just appreciation of the value of medicine as such, or the importance of observing the plainest and most common hygienic rules and conditions, may well be looked upon as a discouraging task. Intelligent men in other matters will astonish us with their ignorance in matters of medicine, and when the various forms of absurdity which now receive credence shall have been exploded and passed away, others will not be wanting to take their places equally absurd and prepostorous. About one quarter of the people follow after and take to the systems of empiricism as they severally make their appearance. Thompsonian, eclectic, galvanic, homeopathic, magnetic, spiritualistic, dermontic, aquatic, etc., each in turn receive their support and is the system they advocate. They are worthy people; in most respects, they have not less common sense and observation than others, but are monomaniac in matters pertaining to medicine. That this is the true condition of things is no disadvantage to the capable physician. On the other hand, we regard it a blessing, since truthful and honest advice, which had a show of consistency and probability about, it would be repudiated by them as too dull and old-fashioned for their faith, having nothing wonderful, mysterious or unaccountable about it, nothing to attract or confound. Physicians do not want such patients, and it cannot be regarded otherwise than a favor that their wants can be supplied from the ever-open fountain of delusion."

The Chairman of the Committee on Membership, Dr. T. M. Johnson, reported that the following named gentlemen had made application and were recommended for membership by the committee: Drs. George H. Westinghouse, E. J. Murphy, W. E. Jennings, Gustav Pohl, H. J. Hill, W. E. Robbins, E. M. Wetherill, E. T Smith and E. T. Stevens.

Upon motion of Dr. J. C. Greene, the report of the committee was accepted and their recommendation adopted.

Dr. Stork, Chairman of the Board of Censors, presented the following report:

### REPORT OF THE BOARD OF CENSORS.

At the last annual meeting of the Society, the following resolutions were passed and referred to the Board of Censors:

1. That the Board be instructed to determine whether a member of this Society can join a Homeopathic Medical Society and still retain his membership in this Society, and whether he is liable for expulsion for such action.

2. That the Board be directed to determine whether a member of this Society dealing in patent medicines and nostrums can hold his membership in this Society.

In regard to the first, the Censors hardly deem themselves competent authority to determine this question. We may say, however, that in this matter "the motive largely determines the morality of the act." If a member of this Society, holding the diploma of a regular medical school, determines to join also a Homeopathic Medical Society, he must have some motive or purpose in doing so, unless he commits such act in aberration of his mind. In this case he could not be held responsible. While your Board deems such conduct of a member highly improper, and while there may be sufficient cause for disciplining him, we also have to admit that a member of this Society has the legal right and the privilege to join other scientific societies, like the Historical, Natural Sciences, or Homeopathic Society, and still retain his membership in the Erie County Medical Society without being liable for expulsion for such action.

Howbeit, if such a case exists in our Society, and the member—as charged in the resolution—should at the present time be of sound mind, his good sense and honor will probably dictate to him to resign his membership, but if he should not do so before the next meeting of the Society, the Secretary should be directed to furnish him with a copy of these proceedings and demand his resignation.

On the second resolution, the Board deems the question rather too vague for opinion. A physician pharmacist might do all named in the resolution without fault and without violating the by-laws and rules of the Society. Many physicians advise and prescribe nostrums and patent or proprietory medicines, and many of the preparations of manufacture of such medicines that are carefully supplied with labels and pamphlets, giving full directions how and for what ailments they are to be used as certain cures, are used by members of the professions, a practice which your Board deems not only highly improper and pernicious, but also very unprofessional.

The Censors are of the opinion that when such *dealing*, as stated in the resolution, becomes an open and distinct medical scandal or heresy, it would come within the meaning of the term "unprofessional conduct."

If, for instance, a member shall undertake to cure incurable diseases, or urge such nostrums and proprietory medicines as infallible remedies, or in any way practice upon the ignorance and credulity of the people to their harm in body or estate, such practice or business, carried on by a member, would be justly considered "*unprofessional,*" and constitute cause for disciplining him. Whether it be sufficient cause for expulsion from the Society—meaning expulsion from the medical profession—your Board is at present unable to determine.

If in this case, as in the other, a member of this Society has made himself guilty of such misconduct, and is at present still engaged in the unprofessional traffic in nostrums and patent medicines, he should feel in honor bound to resign his membership in this Society; if not, direct charges should be preferred against him in proper form.

A resolution was passed some time ago by the Society, by which the Board of Censors was directed to inquire into the law that was passed before the medical law of 1880—a law which provided that no practitioner in a county can be a legal practitioner unless he is a member of a legally-authorized medical society. This question has been before the New York County Medical Society, and the legal opinion there was that the law was still in force, and was not nullified by the law of 1880. Relying upon that legal decision so far, we did not deem it proper to incur the expense of legal advice in this locality; we deemed it unnecessary, as this decision is from very good legal authority in New York, and the legality could only be established by a decision of the courts, if a proper case should be brought before them.

The Board holds that the law that makes it obligatory for a practitioner in a county to apply to a medical society of that county for membership, within thirty days after his residence in that county, is still in force, and unless he does so and becomes a member of a legally-authorized society, he is not a member of the profession in good standing.

Upon motion of Dr. Hopkins, the report of the Board of Censors was received and placed on file.

Upon motion of Dr. Crego, the president then appointed the following nominating committee: Drs. Ring, Davidson, Storck, Folwell and W. D. Greene.

Dr. Lynde moved that the secretary be instructed to send each member of the Society a copy of the proposed amendments to the by-laws, that we may have time to consider them and be prepared to vote upon them at our next regular meeting.

Dr. Coakley reported that the auditing committee found the accounts of the treasurer to be correct.

The following officers, recommended by the nominating committee, were duly elected: Dr. O. C. Strong, President; Dr. J. D. Hill, Vice-President; Dr. William H. Thornton, Secretary; Dr. F. W. Abbott, Treasurer.

Censors: Drs. Edward Storck, H R. Hopkins, W. H. Gail, P. W. VanPeyma and Charles Wetzel.

Committee on Membership: Drs. T. M. Johnson, E. T. Dorland and F. S. Crego.

Dr. Strong addressed the Society in a few well-chosen remarks, acknowledging the honor of his election as President of the Society.

Upon motion, the meeting then adjourned.

Wm. H. Thornton, Secretary.

There were present at the meeting Dr. E. T. Dorland, President; Dr. Thornton, Secretary; Drs. Abbott, Banta, Barnes, Barker, Bourne, Brecht, Briggs, Brown, Coakley, Crego, J. Cronyn, Dagenais, Daggett, Dambach, Daniels, Davidson, L. P Dayton, Dorland, Folwell, Gail, W. D. Greene, J. C. Greene, Gumaer, Haberstro, Hartwig, Harvey, Hill, Hopkins, Howe, F. F. Hoyer, B. Hoyer, Ingraham, Keene, E. H. Long, B. G. Long, Lynde, Meisburger, Nott, O'Brien, Pattison, Prior, Putman, C. A. Ring, Wm. Ring, Samo, Storck, Strong, Walsh, Wyckoff, S. W. Wetmore, Rich, Park, Root, Campbell, Wilson, Porter, Vandenbergh, Bingham, Dwyer, Starr.

